# Impact of early post-pyloric feeding during therapeutic hypothermia on outcomes in patients with traumatic brain injury: a retrospective cohort study

**DOI:** 10.3389/fmed.2026.1924713

**Published:** 2026-09-10

**Authors:** Qiuhong He, Yang Hu, Xiaoli Du, Fengmei Li, Dan Wen, Dan Zhang

**Affiliations:** Intensive Care Unit, Mianyang Central Hospital, School of Medicine, University of Electronic Science and Technology of China, Mianyang, Sichuan, China

**Keywords:** enteral nutrition, feeding intolerance, post-pyloric feeding, therapeutic hypothermia, traumatic brain injury

## Abstract

**Background:**

Although therapeutic hypothermia (TH) is a standard critical care intervention for traumatic brain injury (TBI), it can significantly suppress gastrointestinal motility, leading to feeding intolerance (FI) rates as high as 40%–70%. Post-pyloric feeding (PPF), which bypasses the stomach, offers a potential solution. Nevertheless, the efficacy of PPF in improving outcomes for TBI patients receiving TH has not been established.

**Objective:**

This study aimed to compare the effects of early post-pyloric feeding versus conventional nasogastric tube feeding on neurological outcomes and clinical endpoints in TBI patients undergoing therapeutic hypothermia.

**Methods:**

In this single-center, retrospective cohort study conducted between January 2024 and June 2025, 122 eligible TBI patients were enrolled. Based on the enteral nutrition route established within 48 h of intensive care unit (ICU) admission, patients were divided into a PPF group (*n* = 67) and a nasogastric tube feeding (NGT) group (*n* = 55). All patients received standardized therapeutic hypothermia and nutritional support. The primary outcomes were 30-day neurological outcome and 30-day mortality. Secondary outcomes included the incidence of feeding intolerance, duration of mechanical ventilation, length of ICU stay, and nutritional laboratory indicators.

**Results:**

No statistically significant differences were observed between the PPF and NGT groups in the 30-day favorable neurological outcome rate (29.85% vs. 30.91%, *P* = 0.900) or the 30-day mortality rate (10.44% vs. 7.27%, *P* = 0.582). The incidence of FI was significantly lower in the PPF group (13.43% vs. 30.91%, *P* = 0.019). Additionally, the PPF group had significantly shorter median durations of mechanical ventilation (11 [9–16] days vs. 15 [10–21] days, *P* = 0.012) and ICU stay (13 [11–20] days vs. 20 [13–29] days, *P* = 0.004). No significant differences were identified in nutritional laboratory indicators at the end of TH.

**Conclusion:**

Although early PPF did not significantly improve short-term mortality or neurological outcomes in TBI patients undergoing TH, it effectively reduced the incidence of FI and shortened the duration of mechanical ventilation and ICU stay.

## Background

Traumatic brain injury (TBI) is a leading global cause of disability and mortality (1). Worldwide, there are approximately 70 million new cases of TBI annually (2). In China, the TBI-related mortality rate is about 13 per 100,000 individuals (3). TBI not only causes acute cerebral tissue damage but also exacerbates neurological deterioration through secondary injury mechanisms. Among survivors, approximately 60% experience long-term cognitive, motor, or psychological impairments (4). Therefore, the timely implementation of effective neuroprotective strategies to block or mitigate the secondary injury cascade is crucial for improving the quality of life and functional outcomes in TBI patients.

Therapeutic hypothermia (TH), as a standard critical care intervention, has been widely used in conditions such as hypoxic encephalopathy after cardiac arrest and refractory intracranial hypertension (5, 6). While therapeutic hypothermia remains a clinical practice despite recent RCTs questioning its efficacy regarding mortality and neurological outcomes (7). However therapeutic hypothermia can significantly suppress gastrointestinal function, leading to a sharp increase in the incidence of feeding intolerance (FI) (8). The ESPEN 2023 practical guideline recommends initiating enteral nutrition early in critically ill patients and suggests considering post-pyloric feeding in cases of gastric feeding intolerance (9). Similarly, the ASPEN 2022 guidelines emphasize individualized nutrition therapy and advocate for hypocaloric feeding strategies in specific populations to mitigate metabolic complications (10).

Nasogastric tube feeding (NGT) remains the preferred clinical standard for early nutritional support in TBI patients. However, within the context of hypothermia-induced gastroparesis, the risk of FI with NGT can be as high as 40%–70% (11, 12), often resulting in failure to achieve target caloric and protein intake. The core advantage of post-pyloric feeding (PPF) lies in its ability to bypass the stomach, which is often rendered “paralyzed” by TH, and deliver nutrients directly to the duodenum or jejunum where motility is relatively preserved (13). In critically ill patients at high risk of gastric retention, PPF has been demonstrated to significantly improve feeding tolerance and achievement of nutritional targets compared to intragastric feeding (14, 15). Despite these advantages, the value of PPF, particularly its impact on final patient outcomes, remains a subject of clinical uncertainty, especially within the specific population of TBI patients undergoing TH.

A retrospective cohort study was conducted in the ICU to compare the differences in the rate of favorable neurological outcome and other clinical outcomes between early PPF and NGT in TBI patients undergoing TH.

## Methods

### Study design and participants

This single-center, retrospective cohort study was conducted in the Intensive Care Unit of Mianyang Central Hospital in Sichuan Province, China (Mianyang Critical Care Medical Center, 40 ICU beds) between January 2024 and June 2025. The study protocol was approved by the Hospital Ethics Committee of Mianyang Central Hospital (Approval No.: S20240202-01). As all enrolled participants had impaired consciousness, written informed consent was obtained from their family members.

Patients were divided into a PPF group and a NGT group based on the enteral nutrition support route established within 48 h of ICU admission. The decision to place a nasojejunal tube versus a nasogastric tube was made by the attending physician based on bedside ultrasonographic assessment of gastric motility and residual volume after ICU admission. PPF was preferentially selected when gastric ultrasound indicated severe gastroparesis (gastric antral cross-sectional area > 10 cm^2^ after 6 h of fasting or persistent high gastric residual volume > 200 mL), whereas NGT was the default route when gastric motility was deemed adequate. All tube placements were performed under ultrasound guidance, with final position confirmed by x-ray. The inclusion criteria were as follows: patients with TBI; age ≥ 18 years; underwent standard neurosurgical procedures including decompressive craniectomy, evacuation of intracranial hematomas (epidural, subdural, or intraparenchymal), or intracranial pressure monitor placement as indicated; received TH within 24 h postoperatively; initiation of enteral nutrition within 48 h postoperatively; a Glasgow Coma Scale (GCS) score < 12; and a Nutritional Risk Screening 2002 (NRS 2002) score ≥ 3. Exclusion criteria included conditions contraindicating enteral nutrition, such as gastrointestinal bleeding, intestinal obstruction, or lower gastrointestinal fistula; contraindications for enteral nutrition tube placement, including nasopharyngeal tumors, hemorrhage, or recent surgery; an ICU length of stay < 48 h. Patients were also excluded if they or their family members requested withdrawal of treatment or to exit the study, if the enteral nutrition route was switched or transitioned to parenteral nutrition during the study period, or if their data were incomplete.

### TH

Within 24 h of ICU admission, the patient's core body temperature was maintained at 33–35 °C through intravenous infusion of cold saline and using a temperature-control blanket (Zhejiang Smith Medical Equipment Co., Ltd., China). Core body temperature was monitored via a bladder temperature sensing probe (Shanghai Ruimed Medical Technology Co., Ltd., China). Therapeutic hypothermia was divided into four phases: (1) Induction phase: The goal was to rapidly achieve the target core temperature (33 °C) within 2 h. This was accomplished through intravenous administration of an analgesic (fentanyl) and a sedative (midazolam) to ensure a Shiver Assessment Scale score of 0 and a Richmond Agitation-Sedation Scale (RASS) score of −4 or −5. (2) Maintenance phase: The objective was to maintain the core body temperature between 33 and 34 °C for 24 h. During this phase, serum electrolyte levels were monitored continuously, and sinus rhythm was maintained. The patient's mean arterial pressure was kept at 60–90 mm Hg, arterial oxygen pressure at 70–100 mm Hg, and fasting blood glucose at 110–180 mg/dL. All patients received standardized medical management for intracranial hypertension, specifically intermittent boluses of mannitol (20%, 0.5–1 g/kg) based on intracranial pressure (ICP) monitoring and clinical assessment. (3) Rewarming phase: Rewarming was initiated when imaging examinations indicated reduced cerebral edema and intracranial pressure was below 20 mmHg. The core temperature was gradually increased at a rate of 0.05 °C–0.25 °C per hour, with a target temperature of 36.0 °C–36.5 °C achieved within 48 h. A 3M Bair Hugger Normothermia System forced-air warming blanket was used for the rewarming process. (4) Normothermia maintenance phase: The core temperature was maintained between 36.5 °C and 37.5 °C. To prevent fever, 650 mg of acetaminophen was administered every 6 h via the enteral feeding tube. Given that the peak period of cerebral edema typically occurs 3–5 days after cranial injury (16), the duration of TH was maintained for at least 3–5 days in all patients enrolled in this study.

### Enteral nutrition

For all patients, under ultrasound guidance, either a nasojejunal or nasogastric tube was placed within 24 h of ICU admission, with the position confirmed by x-ray. Enteral nutrition support was initiated within 48 h. All patients received a peptide-based enteral nutrition formulation (Fresenius Kabi Huarui Pharmaceutical Co., Ltd.) via continuous infusion using an enteral feeding pump. The initial infusion rate was set at 50 mL/h and gradually increased to 80–100 mL/h. The energy expenditure (EE) was calculated using a weight-based formula, which included energy calculation [25–30 kcal/(kg·d)] and protein calculation [1.2–2 g/(kg·d)] (17). Hypocaloric nutrition (≤ 70% of EE) was provided during the first 7 days, followed by a gradual increase to full-calorie nutrition thereafter (18). During the enteral nutrition therapy period, all patients were maintained in a semi-recumbent position (30°–45°). They received oral care with chlorhexidine solution every 6–8 h, and blood glucose levels were monitored every 4 h, with a target range of 7.8–10.0 mmol/L. Insulin therapy was initiated when blood glucose levels exceeded 10.0 mmol/L.

### Outcomes

Primary outcomes included 30-day neurological outcomes and 30-day mortality. Neurological outcomes were assessed using the modified Rankin Scale (mRS) at 30 days after symptom onset (19). The mRS is widely used to evaluate global disability and functional outcomes in patients with stroke. Scores range from 0 (no symptoms) to 5 (severe disability), with a score of 6 indicating death. Generally, scores of 0–2 are considered a favorable outcome, while scores of 3–5 indicate an unfavorable outcome. All mRS assessments were performed by attending physicians using a structured protocol to ensure consistency. Secondary endpoints included FI, length of ICU stay, duration of mechanical ventilation, and nutritional laboratory parameters at the end of TH. FI is defined as a composite outcome characterized by the presence of any gastrointestinal symptoms, including gastric retention, diarrhea, constipation, abdominal distension, vomiting/ reflux, and gastrointestinal bleeding.

### Data collection

All data were extracted from the electronic medical record system by trained researchers using a standardized data collection form. Collected patient characteristics included gender, age, body mass index (BMI), Acute Physiology and Chronic Health Evaluation II (APACHE II) score, GCS score, ICU length of stay, and duration of mechanical ventilation. Laboratory data included total protein (TP), albumin (ALB), and hemoglobin (HB) measured at ICU admission and upon completion of TH. Feeding intolerance was assessed during enteral nutrition therapy and diagnosed based on the presence of any gastrointestinal symptoms, including gastric retention (defined as ≥200 mL per 6-hour aspiration or a total daily aspirate volume ≥ 1000 mL), diarrhea (watery or loose stools occurring ≥ 3 times daily with a total stool output ≥ 200 g/day), constipation (no defecation within 72 h or stool output < 50 g), abdominal distension (visible abdominal distension with muscle tension and intra-abdominal pressure > 12 mmHg on two consecutive bladder pressure measurements), vomiting/regurgitation (presence of gastric contents or nutrition formula in the oral cavity or airway tubes), or gastrointestinal bleeding (frank blood in vomit, regurgitant, or stool with a positive occult blood test).

### Statistical analysis

All the data were collected by two independent researchers using a standardized data collection form. Following a dual-verification process, the data were entered into Microsoft Excel 2013. All statistical analyses were subsequently performed using SPSS version 26.0 (IBM Corp., Armonk, NY, USA). Categorical data were described using frequencies and percentages. Normally distributed continuous data were presented as mean ± standard deviation, while non-normally distributed data were expressed as median and interquartile range. Comparisons between groups for continuous variables were performed using the two-tailed t test or Mann–Whitney U test. Categorical variables were compared using the Pearson chi-square test or Fisher's exact test. Prespecified subgroup analyses were conducted based on age, GCS score, and APACHE II score. The *P*-value for interaction was calculated to assess the significance of subgroup differences. All tests were bilateral, with a statistical significance level set at *P* < 0.05.

## Results

### Patient characteristics

**Figure 1:**
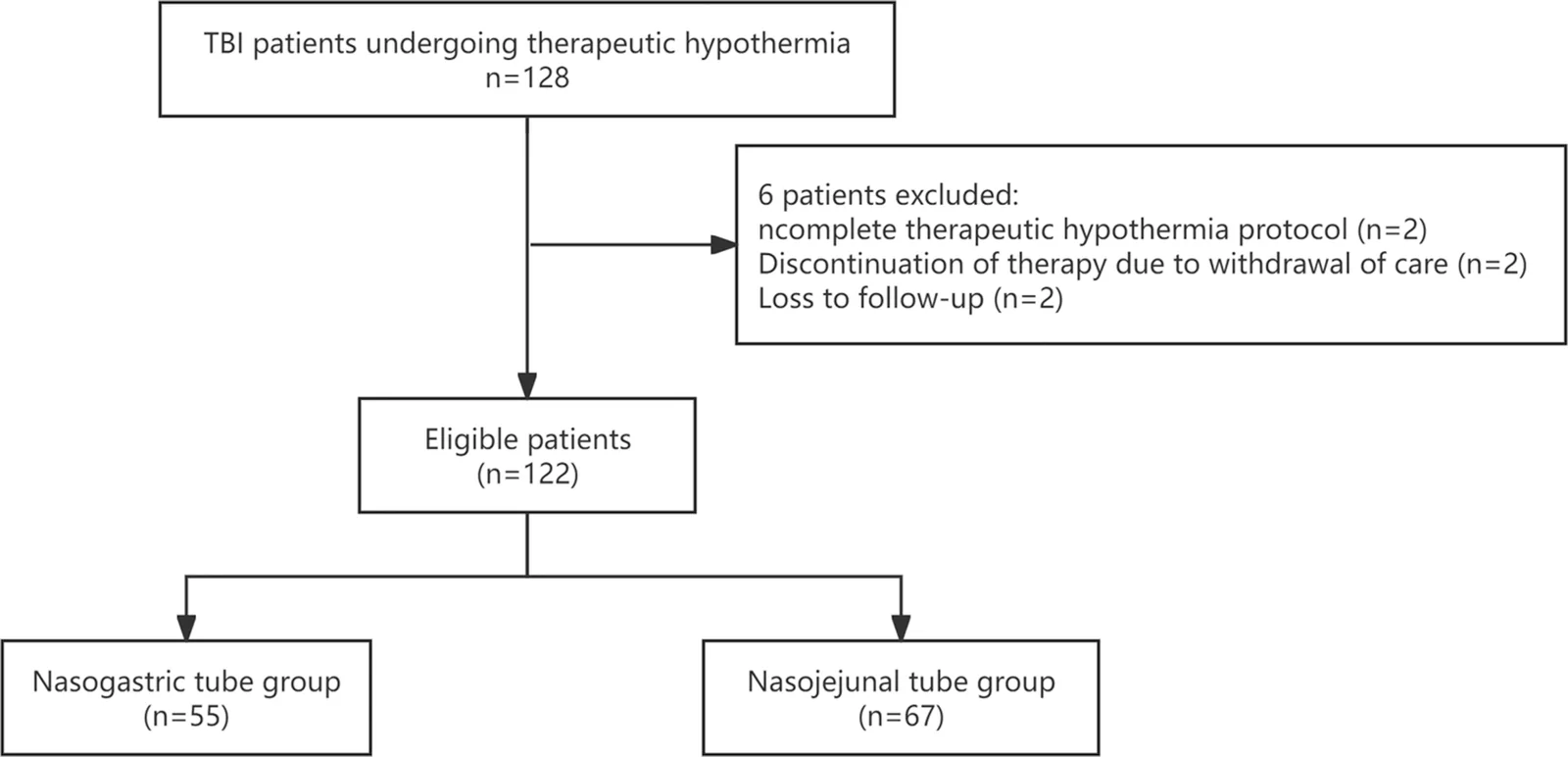
Patient flow diagram.

Of the 128 patients assessed for eligibility, 2 were excluded due to undocumented therapeutic hypothermia management, 2 discontinued intervention following treatment withdrawal, and 2 were lost to follow-up, resulting in 122 patients included in the final analysis (Figure 1). The cohort comprised 55 patients in the NGT group and 67 in the PPF group. No significant differences were observed between the two groups in age, gender, BMI, APACHE II score, GCS score, or nutritional indicators at ICU admission (all *P* > 0.05). The overall population exhibited high APACHE II scores and low GCS scores, likely attributable to the critical nature of their primary conditions requiring postoperative ICU admission, which led to physiological derangements. Additionally, routine postoperative sedation may have further suppressed consciousness, contributing to reduced GCS scores. Baseline characteristics are summarized in Table 1.

**Table 1:** General characteristics of included patients.

Characteristic	NGT (*n* = 55)	PPF (*n* = 67)	Statistical value	*P*
Males, *n* (%)	40 (72.72%)	45 (67.16%)	0.437	0.510
Age, mean (SD), years	56.51 ± 16.79	55.93 ± 13.20	0.215	0.830
BMI, mean (SD)	26.27 ± 3.44	26.55 ± 3.83	−0.427	0.670
APACHE II, median [Q1; Q3]	25 (22,29)	24 (21,28)	−0.740	0.459
GCS, median [Q1; Q3]	5 (3,7)	6 (3,10)	−0.270	0.788
TP at ICU admission, mean (SD), g/L	54.74 ± 9.65	57.47 ± 11.52	−1.401	0.157
ALB at ICU admission, mean (SD), g/L	34.03 ± 6.10	35.48 ± 7.32	−1.175	0.234
HB at ICU admission, mean (SD), g/L	104.49 ± 30.80	114.07 ± 24.28	−1.921	0.063
Surgical procedure, *n* (%)			0.020	0.845
Decompressive craniectomy	28 (50.91%)	35 (52.24%)		
Hematoma evacuation^a^	32 (58.18%)	42 (62.69%)		
ICP monitor placement	41 (74.55%)	48 (71.64%)		

Percentages may add up to >100% as some patients underwent combined procedures.

BMI, body mass index; APACHE II, Acute Physiology and Chronic Health Evaluation II; GCS, Glasgow Coma Scale; ICU, intensive care unit; TP, total protein; ALB, albumin; HB, hemoglobin; NGT, nasogastric tube feeding; PPF, post-pyloric feeding; ICP, Intracranial pressure.

a Includes epidural, subdural, and intraparenchymal hematomas.

### Primary outcomes

**Figure 2:**
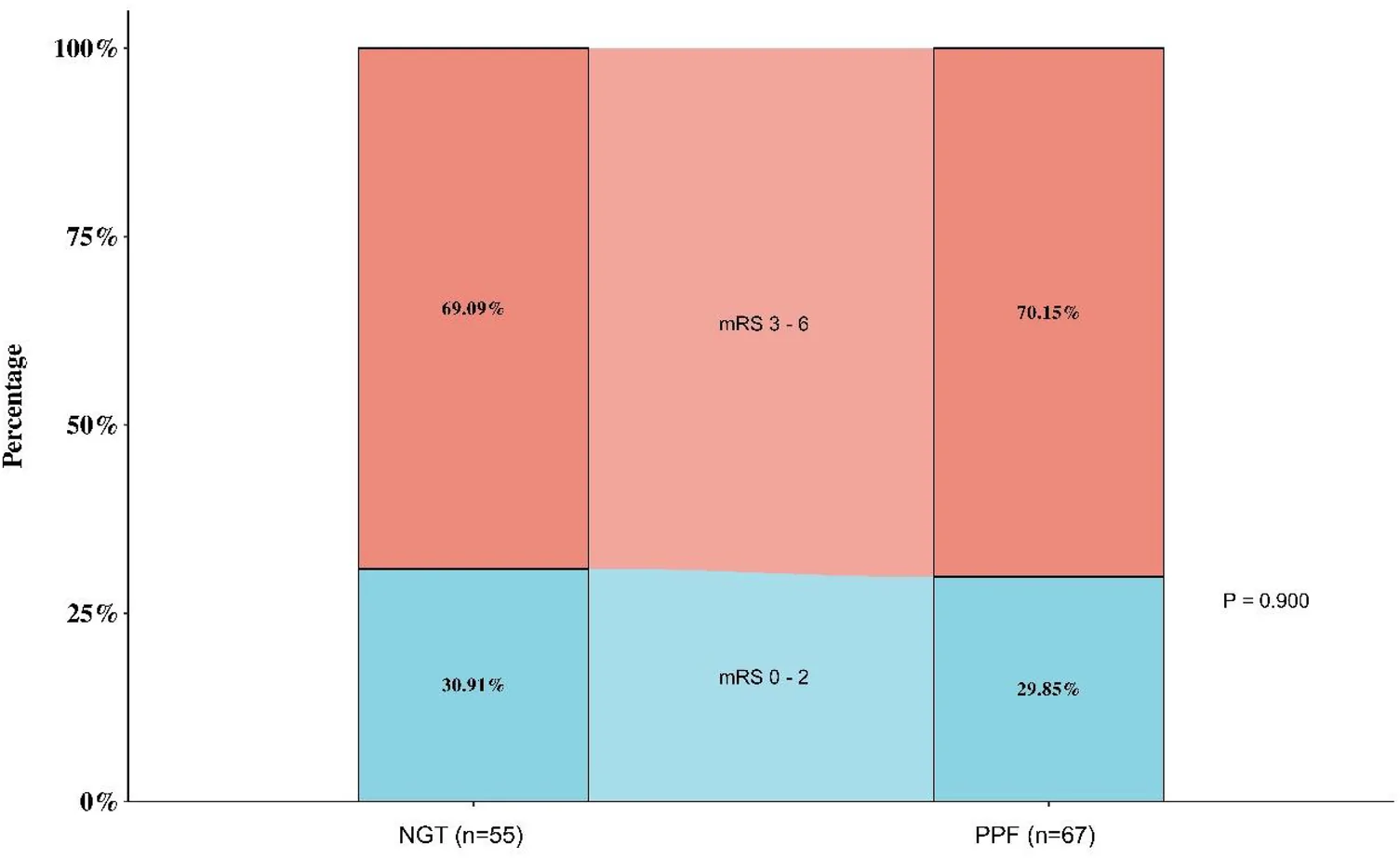
Comparison of neurological favorable outcomes between the two groups. NGT, nasogastric tube feeding; PPF, post-pyloric feeding.

**Figure 3:**
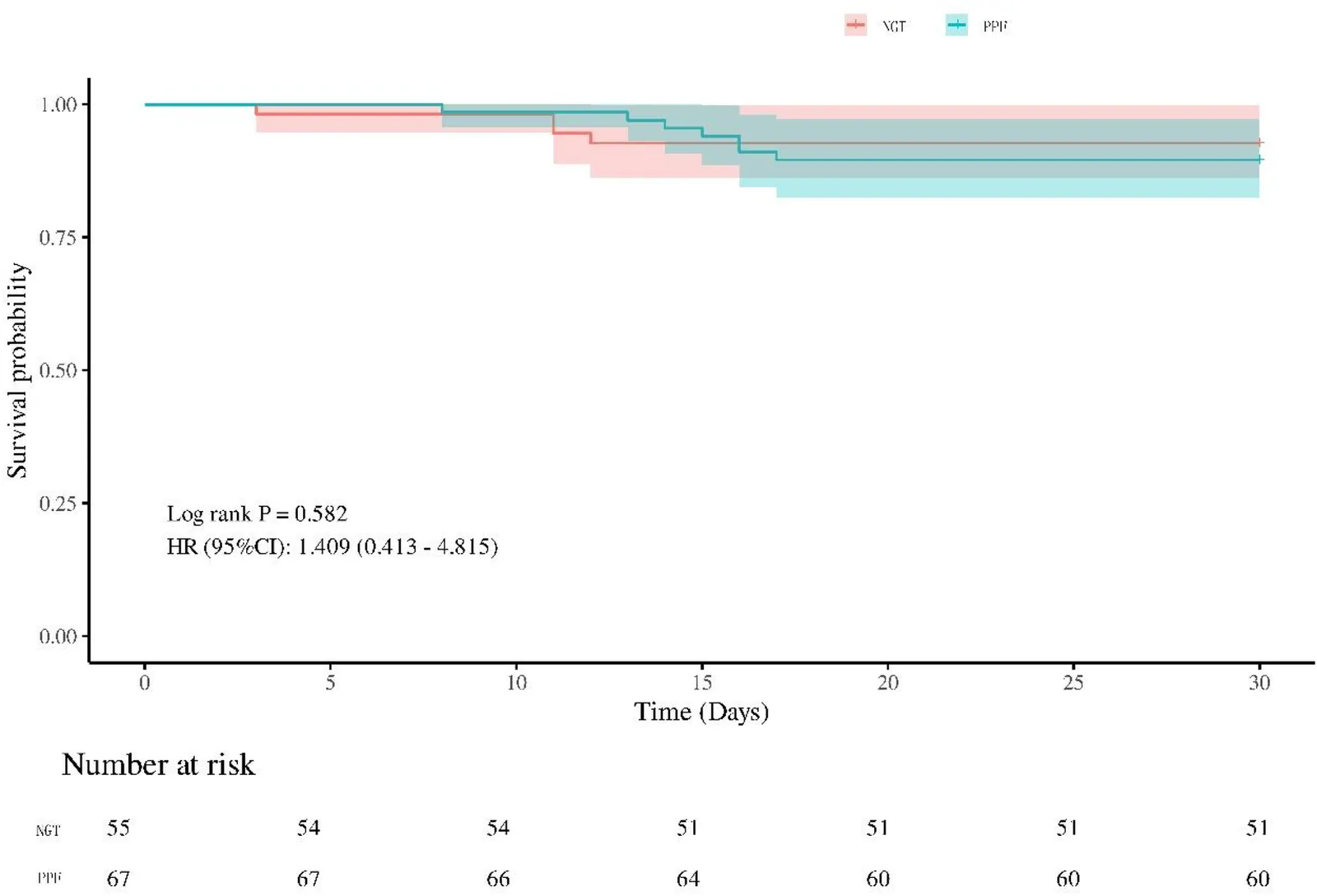
Comparison of 30-day mortality rates between the two groups. NGT, nasogastric tube feeding; PPF, post-pyloric feeding.

Regarding primary outcomes, no statistically significant difference was observed in the 30-day favorable neurological outcome rate between the PPF and NGT groups (29.85% vs. 30.91%, *χ*^2^ = 0.016, *P* = 0.900; Figure 2). Similarly, there was no significant difference in the 30-day mortality rate between the two groups (10.44% vs. 7.27%, 95% CI: 0.413–4.815, *P* = 0.582; Figure 3).

### Secondary outcomes

**Figure 4:**
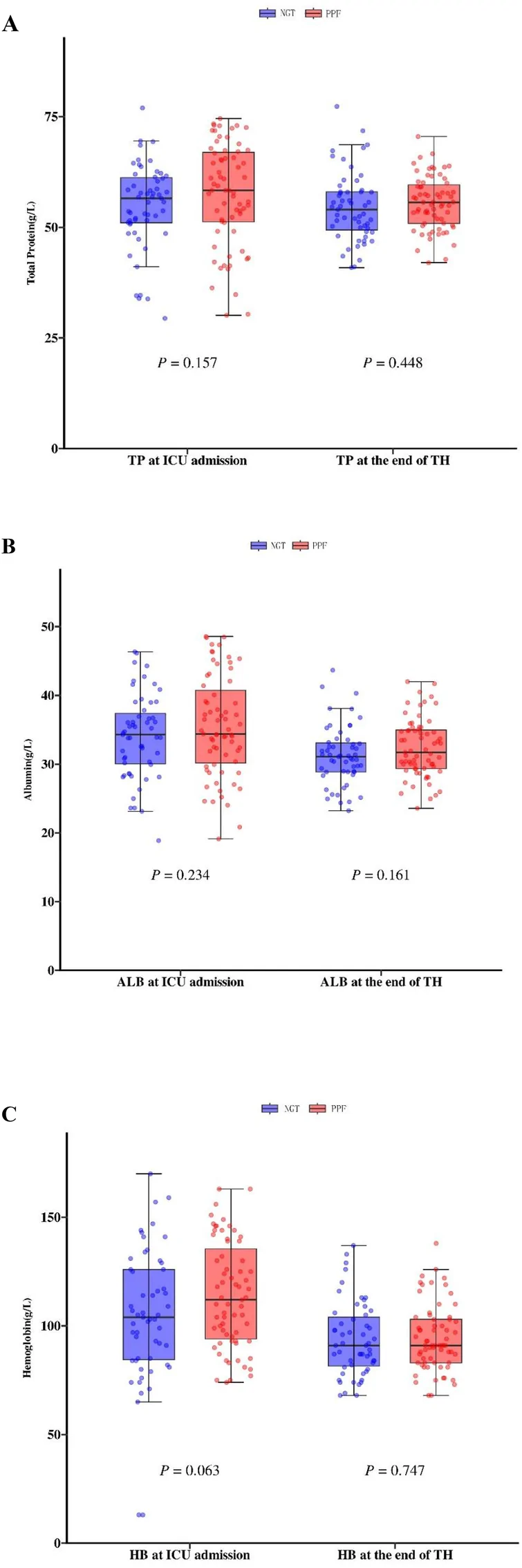
Comparison of nutritional indicators between the two groups at the time of ICU admission and at the end of TH. **(A)** TP, total protein. **(B)** ALB, albumin. **(C)** HB, hemoglobin. NGT, nasogastric tube feeding; PPF, post-pyloric feeding; ICU, intensive care unit; TH, therapeutic hypothermia.

In terms of secondary outcomes, the PPF group demonstrated certain advantages, as summarized in Table 2. The incidence of FI was significantly lower in the PPF group than in the NGT group (13.43% vs. 30.91%, *P* = 0.019). Furthermore, the median duration of mechanical ventilation was significantly shorter in the PPF group compared to the NGT group [11 (9–16) days vs. 15 (10–21) days, *P* = 0.012]. The median length of ICU stay was also significantly reduced in the PPF group [13 (11–20) days vs. 20 (13–29) days, *P* = 0.004]. Regarding nutritional laboratory parameters, no significant differences were observed between the two groups in TP, ALB, or HB levels at the end of TH (Figure 4, all *P* > 0.05).

**Table 2:** Comparison of secondary outcomes between the two groups.

Outcomes	NGT (*n* = 55)	PPF (*n* = 67)	Statistical value	*P*
FI, *n* (%)	17 (30.91%)	9 (13.43%)	5.666	**0.019**
Duration of mechanical ventilation, median [Q1; Q3], day	15 (10,21)	11 (9,16)	−2.499	**0.012**
ICU length of stay, median [Q1; Q3], day	20 (13,29)	13 (11,20)	−2.851	**0.004**
TP at the end of TH, mean (SD), g/L	54.54 ± 7.78	55.51 ± 5.99	−0.781	0.448
ALB at the end of TH, mean (SD), g/L	31.24 ± 4.29	32.34 ± 4.23	−1.412	0.161
HB at the end of TH, mean (SD), g/L	93.55 ± 16.87	94.49 ± 15.14	−0.326	0.747

FI, feeding intolerance; ICU, intensive care unit; TP, total protein; ALB, albumin; HB, hemoglobin; TH, therapeutic hypothermia; NGT, nasogastric tube feeding; PPF, post-pyloric feeding.

Bold values indicates *P* < 0.05.

### Subgroup analysis

**Figure 5:**
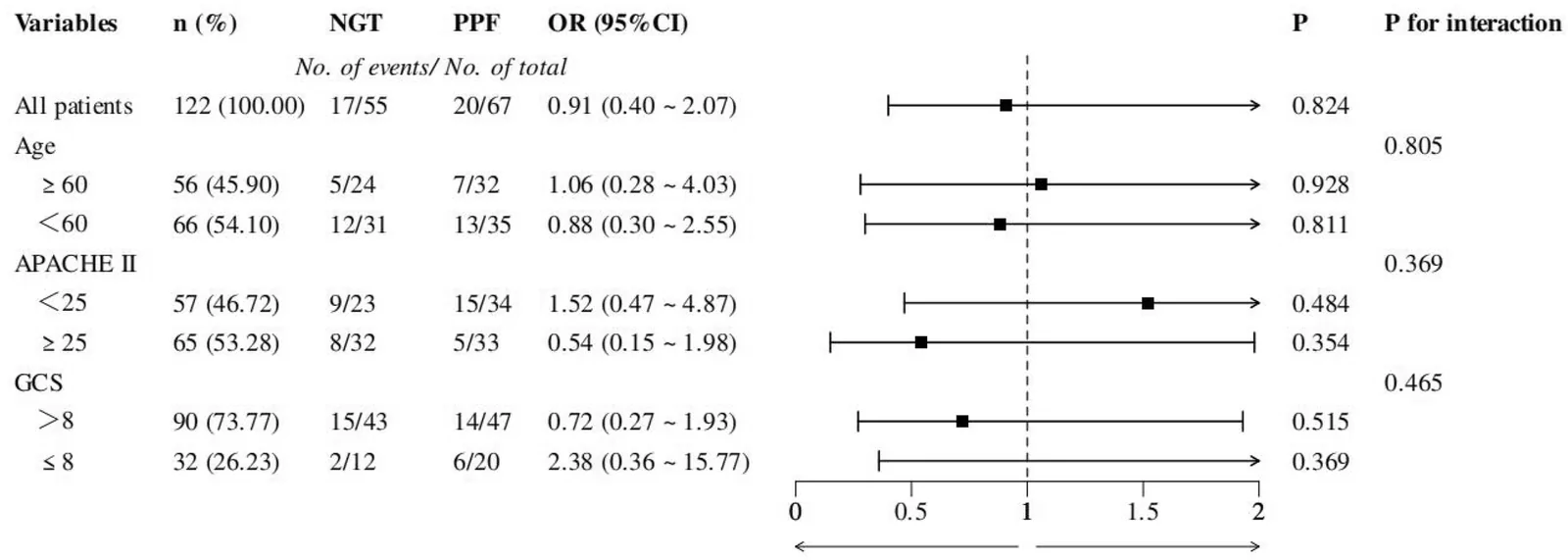
Analysis results of neurological outcomes by age, GCS, and APACHE II subgroups. NGT, nasogastric tube feeding; PPF, post-pyloric feeding; APACHE Ⅱ, Acute Physiology and Chronic Health Evaluation II; GCS, Glasgow Coma Scale.

Subgroup analyses stratified by age (<60 vs. ≥60 years), GCS score (<8 vs. ≥8), and APACHE II score (<25 vs. ≥25) indicated that early PPF did not significantly improve neurological outcomes compared to NGT in any of the predefined subgroups (all *P* > 0.05; Figure 5).

## Discussion

The present retrospective cohort analysis demonstrated that, among TBI patients treated with TH, early initiation of PPF did not yield significant improvements in 30-day mortality (*P* = 0.582) or favorable neurological outcome (*P* = 0.900) when compared to NGT. However, the PPF strategy showed clear advantages in reducing feeding intolerance (*P* = 0.019) and significantly shortening both mechanical ventilation duration (*P* = 0.012) and ICU length of stay (*P* = 0.004).

A key finding of our study was the markedly reduced incidence of FI observed in the PPF group relative to the NGT group (13.43% vs. 30.91%, *P* = 0.019). This outcome is physiologically plausible. Therapeutic hypothermia is known to profoundly inhibit gastrointestinal function, often causing gastroparesis while sparing small bowel motility to a greater extent (20–22). Therefore, the high FI risk associated with conventional intragastric feeding during TH is not unexpected. PPF's core clinical benefit primarily stems from bypassing the hypothermic-induced gastroparesis and delivering nutrients distal to the pylorus (15). Consequently, this approach markedly improves feeding tolerance, allowing for more effective and timely achievement of nutritional goals—an essential component in managing high-consumption critically ill patients (18, 23). Moreover, the decreased frequency of gastric-related complications inherently lowers the risk of aspiration and ventilator-associated pneumonia (15, 24). This protective effect was associated with a significantly reduced median mechanical ventilation duration in the PPF group (11 vs. 15 days, *P* = 0.012). The shorter ventilation duration, alongside reduced gastrointestinal complications, was further associated with a shorter ICU stay (13 vs. 20 days, *P* = 0.004). It should be noted that contemporary guidelines prioritize fever prevention over TH, as high-quality RCTs report no survival benefit from TH in TBI (7). Therefore, the novelty of this study lies not in the effects of TH itself, but in demonstrating that PPF significantly shortens ICU stay, a finding that optimizes resource utilization during TH.

The comparative analysis of primary outcomes indicated that early PPF did not significantly differ from NGT in terms of 30-day mortality (10.44% vs. 7.27%, *P* = 0.582) or favorable neurological outcome (29.85% vs. 30.91%, *P* = 0.900). The findings suggest that the route of nutritional support is not a primary determinant of outcomes in TBI (25). The prognosis for these patients is governed by several more critical factors: first and foremost, the severity of the initial insult and the efficacy of managing secondary injuries (26). Furthermore, the impact of a potent intervention like TH is likely to outweigh the effect of selecting a specific feeding route (27, 28). It is also crucial to recognize that the complexity of neural repair inherently limits what isolated nutritional support can achieve (29, 30). Furthermore, while the 30-day mRS assessment provides an early snapshot of neurological status, it is important to note that functional recovery from TBI is a protracted process extending over months. Therefore, our findings may not reflect the long-term functional trajectory of these patients. Future studies should prioritize extended follow-up periods to capture more definitive functional outcomes. While essential, nutritional support primarily modulates metabolic and immune pathways and cannot directly reverse the structural damage of the primary brain injury (31, 32). Two key limitations should be considered when interpreting our findings. First, the relatively short 30-day follow-up period may not adequately capture the long-term neurological trajectory of TBI patients. Second, the consistent null results observed in all predefined subgroups (age, GCS, and APACHE II) suggest that the neutral effect of PPF on mortality and neurological outcomes is robust across different patient risk categories, rather than being masked by specific population characteristics.

### Limitations

Our study has several limitations that should be considered. First, the absence of *a priori* sample size calculation means the study may be underpowered to detect significant differences in low-frequency events such as mortality or subtle improvements in neurological outcomes. Second, as treatment allocation was clinically driven rather than randomized, residual confounding cannot be entirely excluded despite baseline comparability. Therefore, the observed associations should be interpreted as associative rather than causal. Third, the study lacks critical process indicators. We did not collect or compare the actual achieved targets for caloric and protein intake between the groups, which means our conclusions lack direct supporting data. Also, the 30-day follow-up is insufficient for capturing long-term TBI recovery; future studies should incorporate extended functional assessments. Additionally, the lack of blinding among neurological outcome assessors to the feeding route represents a potential source of assessment bias. Finally, the single-center design and relatively small sample size limit the generalizability of the findings. These conclusions need to be validated in larger-scale, multicenter, prospective studies.

## Conclusion

This study demonstrates that for patients with TBI undergoing TH, early PPF, while not significantly improving 30-day mortality or neurological outcomes compared to NGT, effectively mitigates hypothermia-induced gastrointestinal paralysis, enhances feeding tolerance, and shortens durations of mechanical ventilation and ICU stay. In this specific population, early PPF may be considered a preferable nutritional support strategy to maximize the synergistic benefits of multimodal therapy.

## Data Availability

The original contributions presented in the study are included in the article/Supplementary Material, further inquiries can be directed to the corresponding author.

## References

[1] Taylor CA Bell JM Breiding MJ Xu L. Traumatic brain injury-related emergency department visits, hospitalizations, and deaths - United States, 2007 and 2013. MMWR Surveill Summ. (2017) 66(9):1–16. 10.15585/mmwr.ss6609a1 28301451 PMC5829835

[2] Dewan MC Rattani A Gupta S Baticulon RE Hung Y-C Punchak M. Estimating the global incidence of traumatic brain injury. J Neurosurg. (2019) 130:1080–97. 10.3171/2017.10.JNS17352 29701556

[3] Jiang J-Y Gao G-Y Feng J-F Mao Q Chen L-G Yang X-F. Traumatic brain injury in China. Lancet Neurol. (2019) 18(3):286–95. 10.1016/S1474-4422(18)30469-1 30784557

[4] Capizzi A Woo J Verduzco-Gutierrez M. Traumatic brain injury: an overview of epidemiology, pathophysiology, and medical management. Med Clin North Am. (2020) 104(2):213–38. 10.1016/j.mcna.2019.11.001 32035565

[5] Arrich J Schütz N Oppenauer J Vendt J Holzer M Havel C. Hypothermia for neuroprotection in adults after cardiac arrest. Cochrane Database Syst Rev. (2023) 5(5):CD004128. 10.1002/14651858.CD004128.pub5 37217440 PMC10202224

[6] Moffatt SE. Hypothermia in trauma. Emerg Med J. (2013) 30(12):989–96. 10.1136/emermed-2012-201883 23243045

[7] Cooper DJ Nichol AD Bailey M Bernard S Cameron PA Pili-Floury S. Effect of early sustained prophylactic hypothermia on neurologic outcomes among patients with severe traumatic brain injury: the POLAR randomized clinical trial. JAMA. (2018) 320(21):2211–20. 10.1001/jama.2018.17075 30357266 PMC6583488

[8] Dobak S Rincon F. “Cool” topic: feeding during moderate hypothermia after intracranial hemorrhage. JPEN J Parenter Enteral Nutr. (2017) 41(7):1125–30. 10.1177/0148607116655448 27323775

[9] Singer P Blaser AR Berger MM Calder PC Casaer M Hiesmayr M. ESPEN practical and partially revised guideline: clinical nutrition in the intensive care unit. Clin Nutr. (2023) 42(9):1671–89. 10.1016/j.clnu.2023.07.011 37517372

[10] Compher C Bingham AL McCall M Patel J Rice TW Braunschweig C. Guidelines for the provision of nutrition support therapy in the adult critically ill patient: the American Society for Parenteral and Enteral Nutrition. JPEN J Parenter Enteral Nutr. (2022) 46(1):12–41. 10.1002/jpen.2267 34784064

[11] Hu Y Chen F Xiang X Wang F Hua Z Wei H. Early versus delayed enteral nutrition for neonatal hypoxic-ischemic encephalopathy undergoing therapeutic hypothermia: a randomized controlled trial. Ital J Pediatr. (2022) 48(1):146. 10.1186/s13052-022-01342-2 35971138 PMC9380332

[12] Kumar J Anne RP Meena J Sundaram V Dutta S Kumar P. To feed or not to feed during therapeutic hypothermia in asphyxiated neonates: a systematic review and meta-analysis. Eur J Pediatr. (2023) 182(6):2759–73. 10.1007/s00431-023-04950-0 37014443

[13] Niv E Fireman Z Vaisman N. Post-pyloric feeding. World J Gastroenterol. (2009) 15(11):1281–8. 10.3748/wjg.15.1281 19294757 PMC2658837

[14] Liu Y Wang Y Zhang B Wang J Sun L Xiao Q. Gastric-tube versus post-pyloric feeding in critical patients: a systematic review and meta-analysis of pulmonary aspiration- and nutrition-related outcomes. Eur J Clin Nutr. (2021) 75(9):1337–48. 10.1038/s41430-021-00860-2 33536570

[15] Liu C Jiang J Wen Z You T. Naso-intestinal versus gastric tube for enteral nutrition in patients undergoing mechanical ventilation: a systematic review and meta-analysis. Syst Rev. (2025) 14(1):13. 10.1186/s13643-024-02743-6 39810188 PMC11734493

[16] Nolan JP Sandroni C Cariou A Cronberg T D’Arrigo S Haywood K. European Resuscitation Council and European Society of Intensive Care Medicine guidelines 2025 post-resuscitation care. Resuscitation. (2025) 215 Suppl 1:110809. 10.1016/j.resuscitation.2025.110809 41117575

[17] Zusman O Theilla M Cohen J Kagan I Bendavid I Singer P. Resting energy expenditure, calorie and protein consumption in critically ill patients: a retrospective cohort study. Crit Care. (2016) 20(1):367. 10.1186/s13054-016-1538-4 27832823 PMC5105237

[18] Iapichino G Radrizzani D Armani S Noto A Spanu P Mistraletti G. Metabolic treatment of critically ill patients: energy balance and substrate disposal. Minerva Anestesiol. (2006) 72(6):533–41. 16682927

[19] Van Swieten JC Koudstaal PJ Visser MC Schouten HJ Van Gijn J. Interobserver agreement for the assessment of handicap in stroke patients. Stroke. (1988) 19(5):604–7. 10.1161/01.str.19.5.604 3363593

[20] Nosaka N Okada A Tsukahara H. Effects of therapeutic hypothermia for neuroprotection from the viewpoint of redox regulation. Acta Med Okayama. (2017) 71(1):1–9. 10.18926/AMO/54819 28238004

[21] Bouzat P Payen JF. Therapeutic hypothermia after traumatic brain injury: wrong hypotheses may lead to specious interpretations. Anaesth Crit Care Pain Med. (2019) 38(2):95–6. 10.1016/j.accpm.2019.01.008 30731139

[22] Honeybul S. Reconsidering the role of hypothermia in management of severe traumatic brain injury. J Clin Neurosci. (2016) 28:12–5. 10.1016/j.jocn.2016.01.002 26928159

[23] Wang Y Li Y Li N Li Y Li H Zhang D. Protective nutrition strategy in the acute phase of critical illness: why, what and how to protect. Front Nutr. (2025) 12:1555311. 10.3389/fnut.2025.1555311 40416376 PMC12098084

[24] Alkhawaja S Martin C Butler RJ Gwadry-Sridhar F. Post-pyloric versus gastric tube feeding for preventing pneumonia and improving nutritional outcomes in critically ill adults. Cochrane Database Syst Rev. (2015) 2015(8):CD008875. 10.1002/14651858.CD008875.pub2 26241698 PMC6516803

[25] Maas AIR Menon DK Adelson PD Andelic N Bell MJ Belli A. Traumatic brain injury: integrated approaches to improve prevention, clinical care, and research. Lancet Neurol. (2017) 16(12):987–1048. 10.1016/S1474-4422(17)30371-X 29122524

[26] Maas AIR Fitzgerald M Gao G Gupta D Hutchinson P Manley GT. Traumatic brain injury over the past 20 years: research and clinical progress. Lancet Neurol. (2022) 21(9):768–70. 10.1016/S1474-4422(22)00307-6 35963251

[27] Andrews PJD Sinclair HL Battison CG Polderman KH Citerio G Mascia L. European Society of Intensive Care Medicine Study of therapeutic hypothermia (32–35°C) for intracranial pressure reduction after traumatic brain injury (the Eurotherm3235Trial). Trials. (2011) 12:8. 10.1186/1745-6215-12-8 21226939 PMC3027122

[28] Lin V Tian C Wahlster S Castillo-Pinto C Mainali S Johnson NJ. Temperature control in acute brain injury: an update. Semin Neurol. (2024) 44(3):308–23. 10.1055/s-0044-1785647 38593854

[29] Jassam YN Izzy S Whalen M McGavern DB El Khoury J. Neuroimmunology of traumatic brain injury: time for a paradigm shift. Neuron. (2017) 95(6):1246–65. 10.1016/j.neuron.2017.07.010 28910616 PMC5678753

[30] Simon DW McGeachy MJ Bayır H Clark RS Loane DJ Kochanek PM. The far-reaching scope of neuroinflammation after traumatic brain injury. Nat Rev Neurol. (2017) 13(3):171–91. 10.1038/nrneurol.2017.13 28186177 PMC5675525

[31] Cotoia A Charitos IA Corriero A Tamburrano S Cinnella G. The role of macronutrients and gut microbiota in neuroinflammation post-traumatic brain injury: a narrative review. Nutrients. (2024) 16(24):4359. 10.3390/nu16244359 39770985 PMC11677121

[32] Monti K Conkright MW Eagle SR Lawrence DW Dretsch LM. The role of nutrition in mild traumatic brain injury rehabilitation for service members and veterans. NeuroRehabilitation. (2024) 55(3):281–94. 10.3233/NRE-230241 39269857 PMC11612933

